# Clinical Characteristics as Predictors of Healthcare Resource Utilization in Immunoglobulin A Nephropathy: A Retrospective Database Study

**DOI:** 10.36469/001c.158847

**Published:** 2026-06-08

**Authors:** Nancy Cannizzaro, Hema K. Gandhi, Qiaoling Chen, Sasikiran Nunna, Mohit Mathur, John J. Sim

**Affiliations:** 1 Department of Research & Evaluation Kaiser Permanente Southern California, Pasadena, California; 2 Otsuka Pharmaceutical Development and Commercialization, Inc., Princeton, New Jersey; 3 Visterra, Inc., Waltham, Massachusetts; 4 Division of Nephrology and Hypertension Kaiser Permanente Los Angeles Medical Center, Los Angeles, California; 5 Departments of Health Systems and Clinical Science Kaiser Permanente Bernard J. Tyson School of Medicine, Pasadena, California

**Keywords:** immunoglobulin A nephropathy, nephrology, chronic kidney disease, healthcare resource utilization, progression risk, cost-benefit analysis, comorbidity

## Abstract

**Background:**

Progression rates in immunoglobulin A nephropathy (IgAN) are variable, making future healthcare needs in this patient population difficult to anticipate.

**Objectives:**

We sought to determine whether baseline patient characteristics at biopsy diagnosis are predictive of subsequent healthcare resource utilization (HCRU) in individuals affected by IgAN.

**Methods:**

This was a longitudinal, retrospective cohort study of members enrolled in Kaiser Permanente Southern California, a racially, ethnically, and socioeconomically diverse population. We extracted data on members diagnosed with primary IgAN based on kidney biopsies performed from 2000 to 2021. Statistical associations between indicators of disease severity at baseline and subsequent HCRU were evaluated. The baseline variables assessed were chronic kidney disease (CKD) stage, urine protein creatinine ratio (UPCR), and the individual burden of comorbidities expressed as Elixhauser Comorbidity Index. The HCRU outcomes were emergency department visits, inpatient visits, outpatient visits, radiology, laboratory, and medication dispensations within 2 years after biopsy, all expressed as utilization per patient per month (PPPM).

**Results:**

A total of 612 adults with primary IgAN were identified. Worse baseline CKD stage and UPCR exhibited statistically significant positive correlations with the PPPM for aggregate HCRU. Patients in CKD stage G4 had an overall PPPM of 2.94, which was significantly (P < .01) greater than for each milder CKD category (1.56-2.04). Patients with UPCR >2 g/g had an overall PPPM (2.38), which was significantly (P < .01) greater than each milder UPCR category (1.70-1.84). Aggregate PPPM showed a numerically increasing trend from the least (<0.5 g/g) to most severe UPCR category (>2 g/g). CKD stage, UPCR, and comorbidity score all correlated significantly with individual HCRU outcomes.

**Discussion:**

Easily assessed patient baseline variables including CKD stage, proteinuria, and comorbidities are predictive of HCRU in primary IgAN. Our findings are consistent with recent IgAN management guidelines designating proteinuria ≥0.5 g/g as indicative of elevated progression risk.

**Conclusions:**

Patient clinical characteristics can be used to estimate future HCRU in IgAN, facilitating cost-benefit analysis. The accurate estimation of anticipated HCRU is becoming increasingly important in IgAN as new, disease-specific therapies become available for this long-term, progressive condition.

## INTRODUCTION

Immunoglobulin A nephropathy (IgAN) is the most common primary glomerulonephropathy worldwide, with an overall incidence of at least 2.5 per 100 000 population per year.^1^ The incidence varies by geography, with the highest rates per 100 000 reported among East Asians (1.8-4.2), followed by Europeans (1.0-2.8), and the lowest rate among Africans (0.1).^2^ The global pattern is consistent with differences in the incidence per 100 000 person-years among Asian–Pacific Islander (4.5), White (1.2), and Black (0.6) populations from a study conducted in the United States.^3^ Progression rates vary considerably among affected individuals, but recent cohort data indicate that virtually all patients with IgAN will experience kidney failure during their lifetimes.^4^ Kidney failure imposes the greatest burden of illness on patients and economic costs to the healthcare system.^5^ However, IgAN can exert considerable impacts on patient quality of life and healthcare resource utilization (HCRU) earlier in the course of the disease, well before kidney function decline. Symptoms of pain and fatigue are reported, and the knowledge of having a progressive condition is associated with emotional distress to patients.^6^ Treatment guidelines recommend supportive care with renin-angiotensin system inhibitors in most patients with IgAN to reduce blood pressure and proteinuria and the use of immunosuppressive agents in individuals deemed to be at high risk of rapid progression.^7^ The economic costs associated with immunosuppressive treatment in glomerulonephritis are substantial and have increased over time with the adoption of newer, more expensive agents.^8^

There is a lack of data to quantify the burden of IgAN, including health-economic costs.^6,9^ A number of clinical variables predictive of IgAN progression have been established, including estimated glomerular filtration rate [eGFR], proteinuria, blood pressure, and histopathological classification (ie, MEST-C score),^10^ but the predictive value of baseline patient characteristics for HCRU have not been evaluated to our knowledge. Given that the treatment of IgAN has recently been evolving from supportive measures to disease-specific therapies as novel treatments are introduced, better understanding of which patients are likely to require more intensive management is needed to enable accurate cost-benefit analysis of different treatment options.^10^ To this end, we evaluated data from a diverse cohort of patients with IgAN to identify baseline clinical variables predictive of increased HCRU.

## METHODS

### Design

This was a longitudinal, retrospective cohort study of members enrolled in Kaiser Permanente Southern California (KPSC), a racially, ethnically, and socioeconomically diverse population of over 4.8 million members reflecting the general population of Southern California.^11,12^ The analysis period for the study was January 1, 2000, through November 30, 2022.

### Study Population

For inclusion, members had to be diagnosed with IgAN based on kidney biopsy performed from January 1, 2000, through December 31, 2021, with the date of biopsy serving as the study index date. At least 6 months of continuous membership in KPSC before kidney biopsy were required, in order to reliably capture comorbidities. Exclusion criteria were age <18 years, diagnosis of secondary IgAN, and kidney failure at baseline. Kidney failure was defined as eGFR <15 mL/min/1.73 m^2^ or treatment with hemodialysis, peritoneal dialysis, or kidney transplant.

### Data Collection

Member data were captured in comprehensive electronic health records with information on demographic characteristics, laboratory tests, clinical assessments, diagnostic and procedure codes, and medication use. Extraction methodology for KPSC database analyses has been previously reported.^11,13^ For the present study, kidney biopsy information was obtained from the KPSC Pathology Database by chart review. Patient comorbidities were identified from member records based on *International Classification of Diseases* diagnostic codes, and kidney function parameters calculated from serum creatinine (ie, eGFR) and urinary protein and creatinine values (ie, urine protein creatinine ratio; UPCR). Estimated glomerular filtration rate was derived using the 2021 Chronic Kidney Disease Epidemiology Collaboration Equation.^14^ Information on healthcare resource use within KPSC is captured in the electronic health records, and the research database also includes administrative claims data for out-of-network care.

### Outcomes Assessed

Relationships were evaluated between indicators of disease severity at baseline and subsequent HCRU. The baseline variables assessed were eGFR, UPCR, and burden of comorbidities. The Elixhauser Comorbidity Index was used to assess comorbidities, in which a single score is calculated based on the presence of different comorbidities, and a higher score indicates greater overall burden.^15^

The HCRU outcomes included emergency department (ED) visits, inpatient visits, outpatient visits, radiology, laboratory, and medication dispensations within 2 years after biopsy. All types of HCRU were expressed as utilization per patient per month (PPPM). As an overall measure of HCRU, an aggregate outcome was analyzed that encompassed outpatient, inpatient, and ED visits as well as use of radiology and laboratory services. HCRU was reported as total number of visits/dispenses per resource type per patient during follow-up.

### Statistical Analyses

Baseline clinical characteristics are presented as summary statistics. Aggregate HCRU was compared by baseline kidney disease severity (ie, chronic kidney disease [CKD] stages G1-G4 or UPCR levels <0.5, 0.5 to <1, 1-2, or >2 g/g) using pairwise rankings (Dwass, Steel, Critchlow-Fligner multiple comparison analysis). Associations between baseline disease severity characteristics and HCRU were assessed by negative binomial regression to derive incidence rate ratios (IRR; 95% confidence interval [CI]). IRR were estimated after adjusting for CKD stage G4 (yes vs no), UPCR ≥1 (yes vs no), age (reference: 18-29 years), 30 to 44 years, 45 to 64 years, ≥65 years, sex (male vs female), and Elixhauser Comorbidity Index. All analyses were based on available data; no missing data were imputed.

### Ethical Conduct

This study was approved by the KPSC Institutional Review Board (IRB No. 13750). All the data collected were provided in de-identified and/or anonymized form, and informed consent was not required. The study sponsor was involved in the collection of data, its analysis and interpretation, and/or in the right to approve publication of the finished manuscript.

## RESULTS

### Population

From a population of 12 958 patients who underwent kidney biopsy at KPSC in the years 2000 to 2021, 612 adults with primary IgAN who met the study eligibility criteria were identified (**Supplementary Figure S1**). The population was racially and ethnically diverse and represented a broad spectrum of CKD stages (**Table 1**). Baseline median eGFR was 53.6 mL/min/1.73 m^2^ (interquartile range [IQR], 36.5-79.1) and median UPCR was 1.7 g/g (IQR, 0.9-3.3) The average duration of follow-up was nearly 2 years from time of biopsy, with a mean (standard deviation [SD]) value of 22.3 (4.0) months for the study population. Relative to the groups in CKD stages G1-G3, those in stage G4 tended to be older and numerically more likely to have histories of hypertension, smoking, and cardiometabolic conditions such as diabetes and coronary artery disease.

**Table 1.** Study Population Baseline Characteristics by CKD Stage

	**Total (N = 612)**	**Baseline CKD Stage^d^**			
**Stage G1 (eGFR ≥90) (n = 110)**	**Stage G2 (eGFR 60-89) (n = 152)**	**Stage G3 (eGFR 30-59) (n = 258)**	**Stage G4 (eGFR 15-29) (n = 92)**		
Age, years					
Mean (SD)	45.5 (14.3)	34.2 (10.5)	43.0 (12.8)	49.3 (12.8)	52.1 (15.8)
18-29, n (%)	90 (14.7)	42 (38.2)	23 (15.1)	20 (7.8)	5 (5.4)
30-44, n (%)	229 (37.4)	52 (47.3)	71 (46.7)	77 (29.8)	29 (31.5)
45-64, n (%)	221 (36.1)	14 (12.7)	48 (31.6)	127 (49.2)	32 (34.8)
≥65, n (%)	72 (11.8)	2 (1.8)	10 (6.6)	34 (13.2)	26 (28.3)
Sex, n (%)					
Female	292 (47.7)	75 (68.2)	69 (45.4)	107 (41.5)	41 (44.6)
Male	320 (52.3)	35 (31.8)	83 (54.6)	151 (58.5)	51 (55.4)
Race and ethnicity, n (%)					
Asian/Pacific Islander	189 (30.9)	34 (30.9)	50 (32.9)	78 (30.2)	27 (29.3)
Black	20 (3.3)	2 (1.8)	10 (6.6)	4 (1.6)	4 (4.3)
Hispanic	243 (39.7)	51 (46.4)	57 (37.5)	102 (39.5)	33 (35.9)
White	145 (23.7)	19 (17.3)	31 (20.4)	69 (26.7)	26 (28.3)
Other/unknown	15 (2.5)	4 (3.6)	4 (2.6)	5 (1.9)	2 (2.2)
Systolic blood pressure, mean (SD), mmHg,^a^	128.8 (13.7)	122.3 (11.9)	128. 5 (13.5)	129.8 (13.2)	134.6 (14.8)
Diastolic blood pressure mean (SD), mmHg,^a^	77.0 (9.4)	73.8 (9.3)	78.1 (9.8)	78.0 (8.5)	76.3 (10.5)
BMI, kg/m^2a^					
Mean (SD)	29.1 (6.4)	28.4 (7.7)	29.6 (6.0)	29.0 (5.8)	29.2 (6.8)
Obesity (BMI ≥30), n (%)	205 (33.5)	27 (24.5)	59 (38.8)	84 (32.6)	35 (38.0)
Unknown	50 (8.2)	10 (9.1)	12 (7.9)	17 (6.6)	11 (12.0)
Smoking,^a^ n (%)					
Nonsmoker	419 (68.5)	86 (78.2)	113 (74.3)	168 (65.1)	52 (56.5)
Quit smoking	164 (26.8)	19 (17.3)	30 (19.7)	77 (29.8)	38 (41.3)
Current smoker	21 (3.4)	4 (3.6)	4 (2.6)	11 (4.3)	2 (2.2)
Unknown	8 (1.3)	1 (0.9)	5 (3.3)	2 (0.8)	0 (0.0)
Elixhauser Comorbidity Index^b^					
Mean (SD)	2.9 (2.1)	2.0 (1.9)	2.6 (2.2)	2.9 (1.6)	4.1 (2.5)
Median (IQR)	2.0 (2.0, 4.0)	1.5 (1.0, 3.0)	2.0 (1.0, 3.0)	3.0 (2.0, 4.0)	4.0 (2.0, 5.0)
Hypertension,^b^ n (%)	391 (63.9)	31 (28.2)	85 (55.9)	196 (76.0)	79 (85.9)
Diabetes,^b^ n (%)	81 (13.2)	10 (9.1)	17 (11.2)	37 (14.3)	17 (18.5)
Coronary artery disease,^b^ n (%)	21 (3.4)	2 (1.8)	3 (2.0)	8 (3.1)	8 (8.7)
Heart failure,^b^ n (%)	11 (1.8)	0 (0.0)	2 (1.3)	3 (1.2)	6 (6.5)
Stroke,^b^ n (%)	7 (1.1)	1 (0.9)	0 (0.0)	1 (0.4)	5 (5.4)
Myocardial infarction,^b^ n (%)	6 (1.0)	0 (0.0)	2 (1.3)	3 (1.2)	1 (1.1)
Atrial fibrillation,^b^ n (%)	21 (3.4)	1 (0.9)	7 (4.6)	7 (2.7)	6 (6.5)
Peptic ulcer,^b^ n (%)	7 (1.1)	0 (0.0)	3 (2.0)	2 (0.8)	2 (2.2)
Hematuria,^b^ n (%)	306 (50.0)	73 (66.4)	89 (58.6)	113 (43.8)	31 (33.7)
Baseline UPCR, g/g^c^					
Mean (SD)	2.4 (2.3)	1.7 (1.7)	2.3 (2.3)	2.5 (2.2)	3.3 (2.7)
Median (IQR)	1.7 (0.9, 3.3)	1.3 (0.6, 2.1)	1.5 (0.7, 3.5)	1.8 (1.0, 3.2)	2.6 (1.3, 4.6)
UPCR categories, n (%), g/g					
<0.5	79 (12.9)	21 (19.1)	22 (14.5)	27 (10.5)	9 (9.8)
0.5 to <1	75 (12.3)	16 (14.5)	25 (16.4)	30 (11.6)	4 (4.3)
1–2	173 (28.3)	37 (33.6)	38 (25.0)	81 (31.4)	17 (18.5)
>2	252 (41.2)	29 (26.4)	59 (38.8)	111 (43.0)	53 (57.6)
Unknown	33 (5.4)	7 (6.4)	8 (5.3)	9 (3.5)	9 (9.8)
Albumin, g/dL					
Mean (SD)	3.6 (0.6)	3.7 (0.5)	3.6 (0.6)	3.6 (0.6)	3.4 (0.6)
Median (IQR)	3.7 (3.3, 3.9)	3.7 (3.4, 4.1)	3.7 (3.3, 4.0)	3.7 (3.2, 3.9)	3.5 (3.1, 3.8)
Unknown	84	22	25	27	10
Length of follow-up, mean (SD), months	22.3 (4.0)	22.7 (3.1)	22.2 (4.4)	22.1 (4.2)	22.5 (3.9)

Abbreviations: BMI, body mass index; CKD, chronic kidney disease; eGFR, estimated glomerular filtration rate; IQR, interquartile range; SD, standard deviation; UPCR, urine protein creatinine ratio.

Descriptive statistics were based on available data for continuous variables. Missing data are presented as unknown for categorical variables.

^a^Based on the most recent record within 1 year prior to or as of kidney biopsy.

^b^Comorbidity and medication were based on data within 1 year prior to or as of kidney biopsy; possible range for Elixhauser Comorbidity Index is 0-31.

^c^Record measured closest to kidney biopsy during 1 year before and 30 days after biopsy was retained. Urine albumin-creatinine ratio (n=139) and total urine protein within 24 hours (n = 76) were converted to UPCR by dividing by 0.7 and 1000, respectively.

^d^Measurements at inpatient setting were excluded. GFR within 30 days after biopsy were obtained for 4 patients who had no measurement within 1 year prior to biopsy. eGFR is in mL/min/1.73 m^2^.

### Healthcare Resource Utilization by Kidney Disease Severity

**Figure 1.**
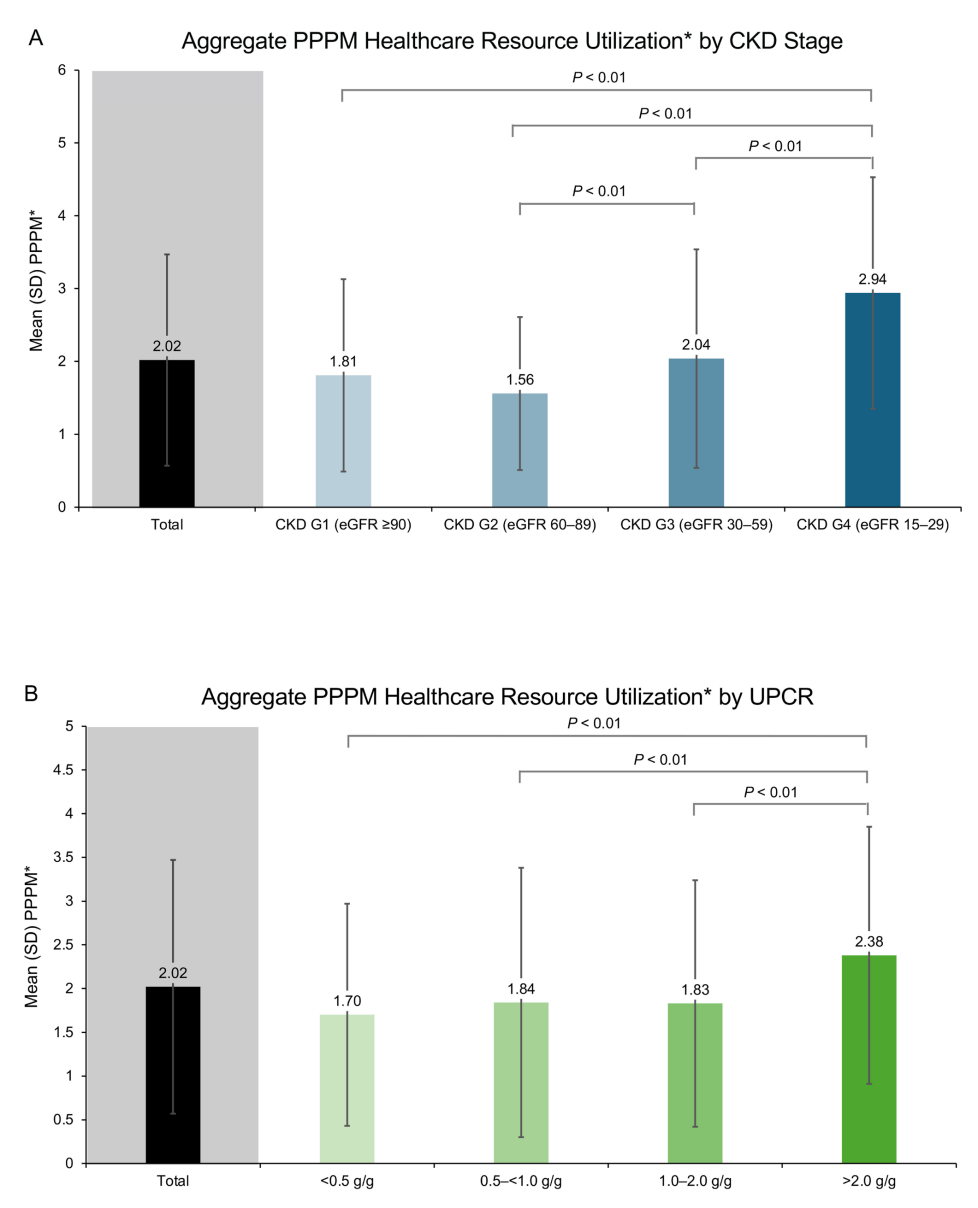
Aggregate Healthcare Resource Utilization (Counts of Visits or Dispensing) by Baseline CKD or UPCR Category Abbreviations: CKD, chronic kidney disease; eGFR, estimated glomerular filtration rate; PPPM, per patient per month; SD, standard deviation; UPCR, urine protein creatinine ratio. *Includes ED visits, outpatient visits, inpatient visits, use of radiology services, and use of laboratory services. CKD and UPCR subgroups were compared using pairwise rankings (Dwass, Steel, Critchlow-Fligner multiple comparison analysis). Significant differences between subgroups are shown. eGFR is in mL/min/1.73 m^2^.

Aggregate HCRU (including outpatient visits, inpatient visits, ED visits, use of radiology services, and use of laboratory services) was significantly greater in patients with worse baseline kidney function (lower eGFR) (**Figure 1A**). A trend of increasing use of healthcare resources was observed by CKD stages. The greatest increased use of healthcare resources was observed between CKD G3 and CKD G4. Furthermore, patients in CKD stage G4 had significantly greater aggregate PPPM utilization than patients at each of the less severe stages (CKD G1-G3). Similarly, for baseline proteinuria, aggregate HCRU was significantly greater in patients with the most severe proteinuria (UPCR >2 g/g) compared with each lesser proteinuria category (**Figure 1B**). Aggregate PPPM exhibited a numerically increasing trend from the least to most severe UPCR category, starting with 0.5 to <1.0 g/g vs <0.5 g/g.

### Regression Analyses

**Figure 2.**
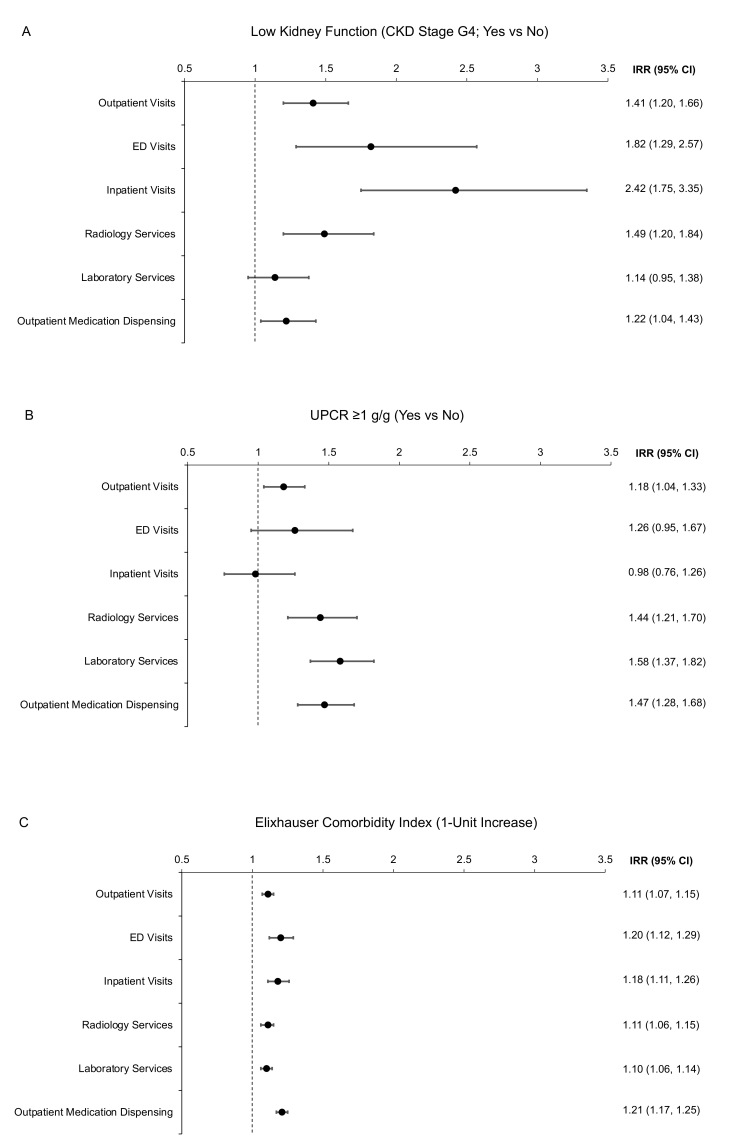
Adjusted Incidence Rate Ratios for Healthcare Resource Utilization by Baseline Disease Severity, as Derived from Negative Binomial Regression Analysis Abbreviations: CI, confidence interval; CKD, chronic kidney disease; ED, emergency department; IRR, incidence rate ratio; UPCR, urine protein creatinine ratio. In the negative binomial regression analyses, for each predictor variable assessed, the other predictor variables were held constant.

In the negative binomial regression analyses adjusted for differences in baseline characteristics, lower kidney function (**Figure 2A**), greater proteinuria (**Figure 2B**), and higher comorbidity burden (**Figure 2C**) were associated with greater likelihood of most types of HCRU. Notably, there was a higher use of inpatient visits among patients with lower kidney function (**Figure 2A**), and more ED visits were made as the UPCR levels increased (**Figure 2B**).

## DISCUSSION

Although there is relatively little research on the individual and social burden of IgAN, existing data suggest that the health-economic cost of managing this condition can be substantial and is likely to increase with disease progression.^6,9^ Our retrospective cohort study adds to the literature by identifying conveniently measured markers of IgAN severity (eGFR, UPCR, and a comorbidity index) that are associated with later HCRU. Given that race and ethnicity are known risk factors for IgAN,^2^ it is important that our findings were demonstrated in a diverse population, as representative of Southern California.

Specifically, our data demonstrated that lower kidney function (CKD stage G4) and greater proteinuria (UPCR >2 g/g) were associated with significantly greater aggregate HCRU over 2 years of follow-up than less severe kidney function impairment and proteinuria. We observed significant differences in PPPM as a surrogate for healthcare utilization. To further contextualize this clinically, a 0.5 increase in PPPM represents 1 additional healthcare event every 2 months. This is an important finding in CKD, where even small rises signal higher disease burden, worsening stability, greater risk of progression, increased hospital and acute care use, higher costs, and declining quality of life.

For proteinuria, HCRU exhibited a numerically increasing trend from least to most severe baseline UPCR, starting with patients at UPCR 0.5 to <1.0 g/g relative to those with UPCR <0.5 g/g. Previous treatment goals targeting proteinuria levels to below 1.0 g/g may impact HCRU in the IgAN population, and our findings support the UPCR goal of <0.5 g/g in the 2025 KDIGO clinical practice guideline, with levels ≥0.5 g/g representing a high-risk population that merits treatment.^7,16^

In the regression analyses, lower kidney function, higher proteinuria, and higher comorbidity burden were all associated with significantly greater risk of specific types of HCRU, such as outpatient visits, laboratory and radiology services, and medication dispenses. These results support the strength of the associations of these baseline markers of disease severity with subsequent utilization.

### Limitations

A limitation of this study is the retrospective design, in which the study cohort was derived from a real-world practice environment. Indication for kidney biopsy was determined by individual practitioners, and thus patients with mild IgAN may not have been biopsied and captured in the study cohort. Additionally, the population was insured and with similar access to care and may not reflect a noninsured population. Therefore, there is an issue with potential selection bias, since our findings may not be representative of those who are not receiving care within an integrated healthcare system.

## CONCLUSIONS

With acknowledgment of the methodological caveats, the results of this study indicate that clinical characteristics at the time of biopsy, such as level of kidney function, severity of proteinuria, and overall burden of comorbidity, predict subsequent HCRU in patients with IgAN. The identification of patients who are more likely to require intensive management in the future can potentially inform the risk/benefit assessments of patients and providers in determining a course of treatment, as well as health-economic decision-making regarding reimbursement for treatment.

### Disclosures

Dr Cannizzaro and Ms Chen report no relationship or financial interest with any entity that would pose a conflict of interest with the subject matter of this article. Dr Gandhi and Dr Nunna are employees of Otsuka Pharmaceutical Development and Commercialization, Inc. Dr Mathur is an employee of Visterra, Inc. Dr Sim reports research grants from Otsuka, Vertex Pharmaceuticals, and Dimerix Bioscience.

## Supplementary Material

Online Supplementary Material

## Data Availability

Data in this manuscript were presented in part at AMCP Nexus 2024; October 14-17, 2024; Las Vegas, Nevada. Editorial services in manuscript preparation were provided by BioScience Communications, Inc. (New York, N.Y.) and funded by Otsuka.
