# Supplementary material for: Clinical Characteristics as Predictors of Healthcare Resource Utilization in Immunoglobulin A Nephropathy: A Retrospective Database Study
Source: J Health Econ Outcomes Res. 2026 Jun 8;13(1):211–7. doi: 10.36469/001c.158847 (PMC13252823; doi:10.36469/001c.158847)
Supplement: Online Supplementary Material [file jheor_2026_13_1_158847_348223.pdf]

Journal of Health Economics and Outcomes Research

Online Supplementary Material

Clinical Characteristics as Predictors of Healthcare Resource Utilization in Immunoglobulin A Nephropathy: A Retrospective Database Study. *JHEOR*. 2026;13(1):211-217. doi:10.36469/jheor.2026.158847

Figure S1: Selection of Study Population

This supplementary material has been provided by the authors to give readers additional information about their work.

This is an open-access article distributed under the terms of the Creative Commons Attribution 4.0 International License (CCBY-4.0). View this license's legal deed at http://creativecommons.org/licenses/by/4.0 and legal code at http://creativecommons.org/licenses/by/4.0/legalcode for more information.

Figure S1. Selection of Study Population

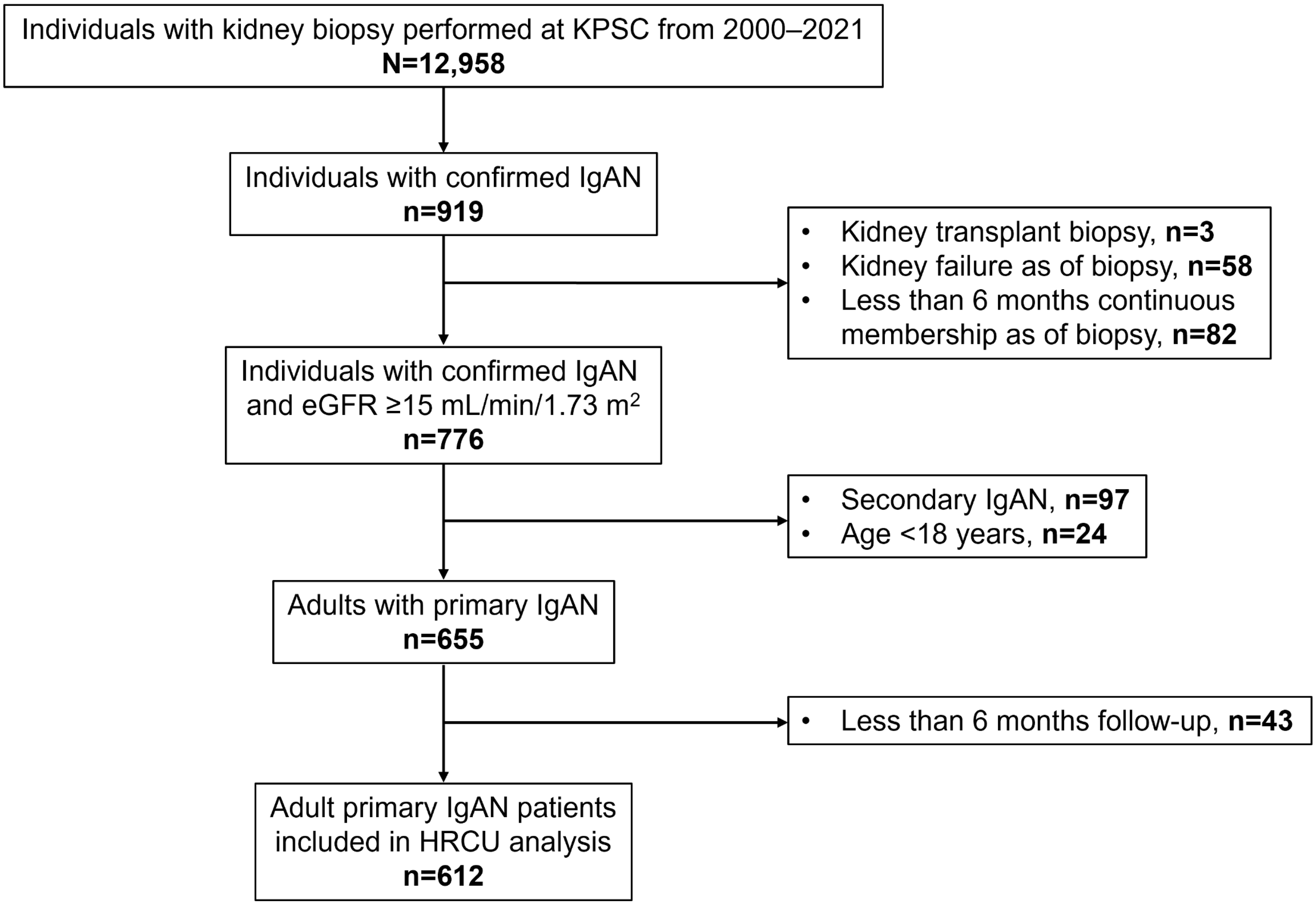

Abbreviations: eGFR, estimated glomerular filtration rate; HCRU, healthcare resource utilization; IgAN, immunoglobulin A nephropathy; KPSC, Kaiser Permanente Southern California.
